# Depth-resolved paired metagenomes and metatranscriptomes from the Lake Erie ‘dead zone’

**DOI:** 10.1128/mra.01013-25

**Published:** 2025-11-05

**Authors:** Katelyn M. Brown, Benjamin F. N. Beall, George S. Bullerjahn, Jacques C. Finlay, Tijana Glavina Del Rio, Gaston E. Small, Robert W. Sterner, R. Michael McKay

**Affiliations:** 1 Great Lakes Institute for Environmental Research, University of Windsor 177440, Windsor, Ontario, Canada; 2 Hatfield Consultants 539490 , North Vancouver, British Columbia, Canada; 3 Department of Biological Sciences, Bowling Green State University 110004, Bowling Green, Ohio, USA; 4 Department of Ecology, Evolution and Behavior, University of Minnesota 172734 , Saint Paul, Minnesota, USA; 5 Department of Energy Joint Genome Institute, Berkeley, California, USA; 6 Biology Department, University of St. Thomas 464711 , Saint Paul, Minnesota, USA; 7 Large Lakes Observatory, University of Minnesota Duluth 14713, Duluth, Minnesota, USA; Montana State University, Bozeman, Montana, USA

**Keywords:** Lake Erie, hypoxia, metagenome, metatranscriptome, greenhouse gas

## Abstract

Metagenomes and metatranscriptomes were generated from the surface mixed layer and hypolimnion at a NOAA-Great Lakes Environmental Research Laboratory Real-Time Coastal Observation Network (ReCON) site in Lake Erie’s central basin during the onset of hypolimnetic hypoxia. Here, we describe the sequencing of the samples, metagenome assembly, and binning of microbial taxa.

## ANNOUNCEMENT

**Fig 1:**
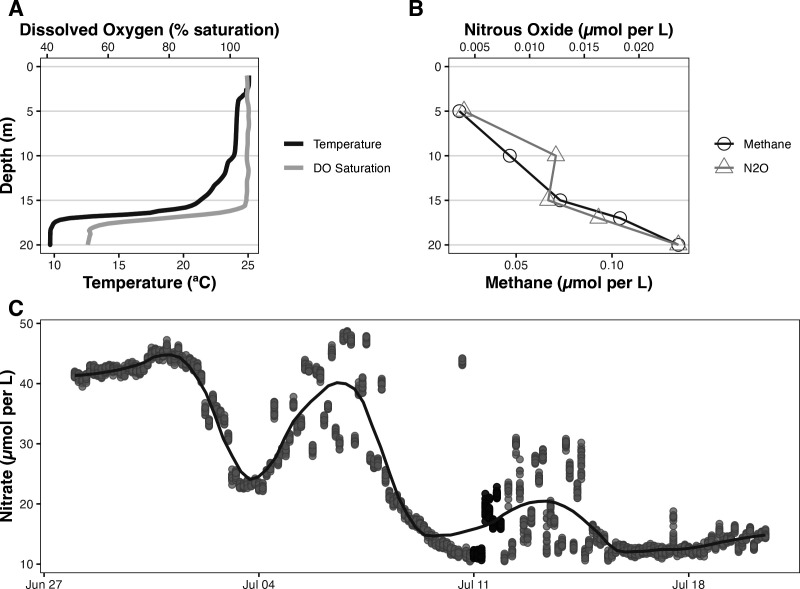
(**A**) The water column at the time of the sample collection was stratified with a warm mixed layer (>24°C) supersaturated with oxygen (105%) overlaying a cooler hypolimnion (9.7°C) depleted in oxygen (53% saturation) (13). (**B**) CH_4_ and N_2_O concentrations at the time of sampling averaged 0.020 and 0.004 µM, respectively, at 5 m and 0.135 and 0.024 µM at 20 m. At this same location, an optical nitrate sensor (*in situ* ultraviolet spectrophotometer [ISUS]; Satlantic, Inc., Halifax, Canada) was deployed at 20 m (**C**). Darkened symbols in panel C represent data points coinciding with the day of metatranscriptome sampling. High-temporal frequency data collection in the hypolimnion (panel C) showed seasonal depletion of nitrate consistent with denitrification.

Lake Erie experiences summertime oxygen depletion, favoring the formation of a hypolimnetic ‘dead zone,’ extending up to 10,000 km^2^ through its central basin (1–4). The reducing environment characteristic of hypoxia threatens drinking water quality (5) and promotes atmospheric greenhouse gas production (e.g., methane [CH_4_], nitrous oxide [N_2_O]) (6–8). Greenhouse gases are more concentrated in the hypolimnion, consistent with microbial transformations in a reducing environment (Fig. 1) (9–12). Omics data from paired epilimnetic and hypolimnetic samples provide insight into biogeochemical cycles during the onset of hypolimnetic hypoxia.

In July 2011, water was collected from the Cleveland Central Buoy ReCON Site (14) in Lake Erie (41.77 N, 81.73 W) at 5 (epilimnion) and 20 m (hypolimnion) depth. The site was stratified with distinct physico-chemical characteristics between the layers (Fig. 1). Depth-resolved biomass was concentrated on Sterivex cartridge filters (0.22 µm; Sigma Aldrich, St. Louis, MO) and frozen in liquid nitrogen. For DNA, cartridges were incubated (1 h) at room temperature with 2 mL of 0.4× STET buffer containing lysozyme (10 mg mL^−1^) and extracted following reference 15. For RNA, 2 mL of the RNAprotect bacteria reagent (Qiagen, Germantown, MD) was passed through the cartridge, then 400 µL of TE buffer containing 15 mg mL^−1^ lysozyme was added. After a 10 min incubation vortexing every 2 min, 1.4 mL of RNA lysis buffer (Qiagen) was added, and cartridges were incubated for 1 h. The solution was transferred to a 15 mL conical tube and extracted using the RNeasy Mini Kit (Qiagen). DNase digestion was completed on-column with RNase-free DNase (Qiagen). Extracted nucleic acids were stored at −80°C.

Extracted nucleic acids were sequenced at the Joint Genome Institute (JGI; Berkeley, CA). For RNA sequencing, rRNAs were depleted (Ribo-Zero rRNA Removal Kit, Illumina, San Diego, CA), and cDNA libraries were generated (TruSeq Stranded mRNA Kit, Illumina). DNA libraries were prepared using a KAPA-Illumina Library Creation Kit (Roche, Basel, Switzerland). Libraries were sequenced on a HiSeq 2000 (2 × 150 bp). Metagenomic libraries contained 128,422,566 (epilimnion) and 140,894,230 (hypolimnion) reads, and metatranscriptomic libraries contained 109,241,050 (epilimnion) and 111,257,782 (hypolimnion) reads. Default parameters were used, unless otherwise noted. Metagenomic reads were error-corrected with bfc (v.r181; kmer = 21; 16) and assembled with SPAdes (v.3.10.0-dev; --meta --only-assembler -k 21,33,55,77,99,127; 17). Filtered reads were mapped to the assembly with bwa (v.0.7.15-r1142-dirty; 18). Contaminant contigs were identified by mapping to the JGI eukaryotic contaminant database (BBMap, v.35.85) and removed if there was ≥90% identity match. Binning was completed with MetaBAT v.0.32.4 (19), and bin quality was assessed with CheckM v.1.2.2 (20), then classified with the Genome Taxonomy Database (v.86) and GTDB-Tk v.0.1.6 (21).

Assembly of the epilimnion metagenomic reads yielded 1,167,820 contigs and N_50_ of 232,621, and the hypolimnion assembly had 1,527,872 contigs and N_50_ of 277,434. There were 17 and 16 medium quality bins from the epilimnion and hypolimnion samples, respectively (22). In the epilimnion, two bins (*Proteobacteria*) had a coverage greater than 100×. The hypolimnion had one bin (*Chitinophagaceae*, *Bacteroidota*) with coverage greater than 100× (Table 1).

**TABLE 1:** Summary of the microbial bins from the metagenomic epilimnion and hypolimnion samples at the Cleveland Central Buoy Site

Bin ID	GTDB taxonomy lineage	Bin completeness (%)	Bin contamination (%)	Average coverage (×)	Size (Mb)	Gene count	No. contigs	Gc (%)	N_50_
Epilimnion									
3300027720_26	*Bacteria*; *Proteobacteria*; *Gammaproteobacteria*; *Betaproteobacteriales*; *Burkholderiaceae; Polynucleobacter*; GCA_002292975.1	70.54	2.27	136.18	1.26	1,366	139	46.22	11,474
3300027720_34	*Bacteria*; *Proteobacteria*; *Gammaproteobacteria*; *Betaproteobacteriales*; *Methylophilaceae; Methylopumilus*; *Methylopumilus planktonicus*	80.24	2.61	119.18	1.03	1,165	95	37.00	19,215
3300027720_19	*Bacteria*; *Verrucomicrobiota*; *Verrucomicrobiae*; *Opitutales*; UBA953; UBA953	74.01	1.72	82.56	1.39	1,414	209	63.94	6,822
3300027720_21	*Bacteria*; *Bacteroidota*; *Bacteroidia*; *Chitinophagales*; *Chitinophagaceae*	56.95	0.18	28.78	1.38	1,372	255	44.33	5,328
3300027720_36	*Bacteria*; *Actinobacteriota*; *Actinobacteria*; *Nanopelagicales*; *Nanopelagicaceae*; *Planktophila*	57.22	0.26	22.61	0.93	1,051	142	46.41	6,722
3300027720_24	*Bacteria*; *Proteobacteria*; *Gammaproteobacteria*; *Betaproteobacteriales*; *Burkholderiaceae*; *Polaromonas*	51.46	2.80	21.08	1.29	1,520	254	59.87	5,292
3300027720_5	*Bacteria*; *Verrucomicrobiota*; *Verrucomicrobiae*; *Pedosphaerales*; UBA9464; UBA9464	82.81	1.55	20.92	3.52	3,100	457	60.13	9,140
3300027720_32	*Bacteria*; *Verrucomicrobiota*; *Verrucomicrobiae*; *Opitutales*; UBA953; UBA953	70.21	1.01	20.07	1.13	1,163	161	48.22	7,359
3300027720_40	*Bacteria*; *Chloroflexota*; Ellin6529; CSP1-4; UBA10416; UBA10416	71.30	0.93	19.19	0.86	969	128	63.57	7,743
3300027720_17	*Bacteria*; *Actinobacteriota*; *Acidimicrobiia*; *Microtrichales*; *Ilumatobacteraceae*; UBA3006	79.74	7.91	17.48	1.48	1,692	202	51.69	8,644
3300027720_3	*Bacteria*; *Verrucomicrobiota*; *Verrucomicrobiae*; *Opitutales*; *Opitutaceae*; Tous-C4FEB	87.85	2.40	16.83	3.44	3,191	398	62.79	10,213
3300027720_4	*Bacteria; Proteobacteria; Gammaproteobacteria; Betaproteobacteriales; Burkholderiaceae; Aquabacterium*	92.66	5.48	16.79	3.58	3,426	313	66.28	15,615
3300027720_16	*Bacteria*; *Verrucomicrobiota*; *Verrucomicrobiae*; *Opitutales*; UBA953; UBA953	67.11	1.35	16.75	1.57	1,514	188	60.75	10,314
3300027720_29	*Bacteria*; *Bacteroidota*; *Bacteroidia*; *Chitinophagales*; *Chitinophagaceae*; UBA10320	52.13	2.09	13.88	1.23	1,319	237	33.51	5,293
3300027720_9	*Bacteria*; *Planctomycetota*; *Phycisphaerae*; *Phycisphaerales*; SM1A02	75.50	2.50	12.72	2.64	2,380	412	61.91	6,984
3300027720_11	*Bacteria*; *Bacteroidota*; *Bacteroidia*; *Flavobacteriales*; UA16; UBA4660	81.27	1.09	11.34	2.43	2,234	313	48.39	9,035
3300027720_20	*Bacteria*; *Bacteroidota*; *Bacteroidia*; *Chitinophagales*; *Chitinophagaceae*; UBA8137	52.96	1.15	10.28	1.37	1,386	240	37.99	5,804
Hypolimnion									
3300027797_29	*Bacteria*; *Bacteroidota*; *Bacteroidia*; *Chitinophagales*; *Chitinophagaceae*; UBA8137	62.93	1.72	129.53	1.46	1,469	230	35.45	6,804
3300027797_39	*Bacteria*; *Actinobacteriota*; *Acidimicrobiia*; *Microtrichales*; *Ilumatobacteraceae*; UBA3006	63.68	1.85	72.67	1.18	1,358	174	51.90	7,391
3300027797_13	*Bacteria*; *Verrucomicrobiota*; *Verrucomicrobiae*; *Pedosphaerales*; UBA9464; UBA9464	60.94	0.14	65.36	2.09	1,970	412	60.04	4,892
3300027797_68	*Bacteria*; *Chloroflexota*; Ellin6529; CSP1-4; UBA10416; UBA10416	51.52	8.33	42.52	0.67	771	153	63.69	4,327
3300027797_14	*Bacteria*; *Verrucomicrobiota*; *Verrucomicrobiae*; *Opitutales*; UBA953; UBA953	81.07	7.06	41.71	1.88	1,852	258	67.75	8,483
3300027797_38	*Bacteria*; *Verrucomicrobiota*; *Verrucomicrobiae*; *Opitutales*; *Opitutaceae*; *Lacunisphaera*; GCA_002304425.1	51.20	0.00	35.51	1.18	1,220	251	56.97	4,614
3300027797_51	*Bacteria*; *Proteobacteria*; *Alphaproteobacteria*; *Rickettsiales*; UBA4311	65.96	1.42	32.25	0.86	875	101	38.22	9,810
3300027797_19	*Bacteria*; *Cyanobacteriota*; *Cyanobacteriia*; *Synechococcales_A*; *Cyanobiaceae*; *Cyanobium*	80.39	4.60	28.58	1.76	2,031	273	65.48	7,089
3300027797_30	*Bacteria*; *Bacteroidota*; *Bacteroidia*; *Sphingobacteriales*; *Sphingobacteriaceae*	79.77	2.18	27.63	1.42	1,373	38	39.98	63,889
3300027797_6	*Bacteria*; *Proteobacteria*; *Gammaproteobacteria*; *Betaproteobacteriales*; *Burkholderiaceae*; *Aquabacterium*	99.32	1.92	25.25	3.93	3,645	189	67.12	32,300
3300027797_28	*Bacteria*; *Bacteroidota*; *Bacteroidia*; *Cytophagales*; *Cyclobacteriaceae*; *Algoriphagus*	56.78	3.38	21.59	1.49	1,600	303	44.48	4,839
3300027797_35	*Bacteria*; *Bacteroidota*; *Bacteroidia*; *Chitinophagales*; *Chitinophagaceae*	54.56	0.90	16.30	1.34	1,321	246	44.54	5,455
3300027797_18	*Bacteria*; *Proteobacteria*; *Gammaproteobacteria*; *Steroidobacterales*; *Steroidobacteraceae*; UBA964	76.27	0.82	14.74	1.74	1,811	252	66.29	7,981
3300027797_50	*Bacteria*; *Actinobacteriota*; *Actinobacteria*; *Nanopelagicales*; *Nanopelagicaceae*; *Planktophila*	54.34	1.72	14.30	0.85	978	174	45.14	4,905
3300027797_15	*Bacteria*; *Bacteroidota*; *Bacteroidia*; *Chitinophagales*	54.54	6.87	10.25	1.82	1,903	390	36.80	4,625
3300027797_33	*Bacteria*; *Bacteroidota*; *Bacteroidia*; *Flavobacteriales*; *Crocinitomicaceae*; UBA952	51.84	3.57	9.26	1.36	1,446	261	41.27	5,110

## Data Availability

The raw reads for the Cleveland Central Buoy Site have been deposited in the NCBI Sequence Read Archive under accession numbers SRX3311440 (epilimnion) and SRX3311442 (hypolimnion). Raw metatranscriptomic reads have been deposited under accession numbers SRX3311909 (epilimnion) and SRX3311907 (hypolimnion). Assembled metagenomic contigs are available through the JGI Integrated Microbial Genomes and Microbiomes (IMG) database under Taxon Object IDs 3300027720 (epilimnion) and 3300027797 (hypolimnion). Microbial bins are also available through the JGI IMG database using bin IDs found in Table 1. Additional physicochemical data to support the sequencing project are found in the NSF Biological and Chemical Oceanography Data Management Office (BCO-DMO) database as datasetdata set 3632.
